# Sarcopenia is Related to Mortality in the Acutely Hospitalized Geriatric Patient

**DOI:** 10.1007/s12603-018-1134-1

**Published:** 2019-01-29

**Authors:** Walther M. W. H. Sipers, W. de Blois, J. M. G. A. Schols, L. J. C. van Loon, Lex B. Verdijk

**Affiliations:** 1 Department of Geriatric Medicine, Zuyderland Medical Center, P.O. Box 5500, 6130 MB Sittard-Geleen, The Netherlands; 2 Department of Human Biology, NUTRIM School of Nutrition and Translational Research in Metabolism Maastricht University, Maastricht, The Netherlands; 3 Department of Health Services Research and Department of Family Medicine, CAPHRI, Maastricht University, Maastricht, The Netherlands; 4 Department of Human Biology, Maastricht University Centre, P.O. Box 616, 6200 MD Maastricht, The Netherlands

**Keywords:** Gait speed, skeletal muscle mass, fat mass, phase angle, sarcopenia

## Abstract

**Background:**

Sarcopenia is defined as low skeletal muscle mass with poor physical performance, representing a strong prognostic factor for mortality in older people. Although highly prevalent in hospitalized geriatric patients, it is unknown whether sarcopenia can also predict mortality in these patients.

**Objective:**

To determine the association between sarcopenia according the criteria of the European Working Group on Sarcopenia in Older People (EWGSOP), International Working Group on Sarcopenia (IWGS), Special Interest Group of Sarcopenia, Cachexia and Wasting Disorders (SIG) and Foundation for the National Institutes of Health (FNIH) and 2-year mortality in acutely hospitalized geriatric patients.

**Design:**

81 patients (84±5 y) admitted to the acute geriatric ward participated in this study. Body composition assessment (bio-impedance, Maltron Bioscan 920-II) and physical performance tests were performed, and mortality information was retrieved through patient files.

**Results:**

Prevalence rates of sarcopenia were 51% (EWGSOP), 75% (IWGS), 69% (SIG), and 27% (FNIH). Based on Cox proportional hazard ratio (HR) analysis, 2-year mortality was significantly higher in sarcopenic patients versus non-sarcopenic patients when using the EWGSOP (2-y: HR 4.310; CI-95%:2.092- 8.850; *P*<0.001) and FNIH criteria (2-y: HR 3.571; CI-95%:1.901-6.711; *P*<0.001). Skeletal muscle mass index, fat mass index, body mass index, phase angle and gait speed were significantly lower in the geriatric patients who deceased after 2 years versus those who were still alive. Cox proportional HR analyses showed that higher phase angle (HR 0.678; CI-95%:0.531- 0.864; P=0.002) and higher fat mass index (HR 0.839; CI-95%:0.758-0.928; P=0.001) significantly reduced 2-y mortality probability. Combining sarcopenia criteria and separate patient characteristics finally resulted in a model in which HRs for sarcopenia (EWGSOP and FNIH) as well as phase angle significantly predicted mortality probability.

**Conclusion:**

Sarcopenia is prevalent in acutely hospitalized geriatric patients and is associated with significantly higher 2-year mortality according the EWGSOP and FNIH criteria.

**Electronic Supplementary Material:**

Supplementary material is available for this article at 10.1007/s12603-018-1134-1 and is accessible for authorized users.

## Introduction

Aging is associated with progressive loss of skeletal muscle mass and strength, commonly termed sarcopenia (1, 2). This age-related decline in skeletal muscle mass and strength impairs functional performance, leading to a decreased level of independence and an increased mortality (3-5). Prevalence of sarcopenia varies widely and depends on the definition, the population studied, and the methodology used for measuring different domains of sarcopenia such as muscle mass, gait speed and grip strength. Hence, sarcopenia prevalence in community dwelling older people has been estimated at 5.3% for men and 13.3% for women according to the European Working Group on Sarcopenia in Older People (EWGSOP) definition; 5.1% for men and 11.8% for women according to the International Working Group on Sarcopenia (IWGS) definition; and 1.3% for men and 2.3% for women according to the Foundation for the National Institutes of Health (FNIH) criteria (6, 7). Furthermore, prevalence tends to be higher in acute care hospital settings (1, 8) with the highest prevalence in hospitalized geriatric patients, ranging between 21-46% (9-13).

Handgrip strength, gait speed and skeletal muscle mass are key features in the operational definition of the EWGSOP and FNIH, however they differ in cut off values and techniques used to assess muscle mass. The IWGS and Special Interest Group of Sarcopenia, Cachexia and Wasting Disorders (SIG) criteria of sarcopenia only incorporate gait speed and muscle mass as key features, although again with different cut off values. While there is strong agreement between the different criteria for the “no sarcopenia” situation, the percent agreement for the classification “sarcopenia” appears rather low, ranging from 5 to 32 % (14). Nonetheless, sarcopenia in older people in the community is associated with increased risk of incident disability, institutionalization, and mortality; independent of whether it is defined by the EWGSOP (1), IWGS (15), or FNIH criteria (4, 6, 16). Despite its strong prognostic value for mortality in community-dwelling elderly, and the high prevalence of sarcopenia in hospitalized geriatric patients, it is unclear to what extent sarcopenia is also associated with mortality in the hospitalized geriatric patient. The study of Cerri and colleagues (12) and Perez-Zepeda and co-workers (13) are currently the only two studies concerning mortality in sarcopenic geriatric patients admitted to respectively an acute geriatric ward and a Geriatric Management and Evaluation Unit. In their work, sarcopenia was diagnosed in 21.4% of 103 (12) and 40.1% of 172 (13) geriatric patients using the EWGSOP definition. In both studies more patients had deceased in the sarcopenic versus non-sarcopenic group following 3 (12) and 12 months of follow-up (12, 13), suggesting that sarcopenia in acutely ill geriatric patients may indeed be associated with increased mortality. Knowledge about mortality risk could be of value in targeting medical treatment in relation to hospitalized geriatric patients with limited life expectancy. However, there are no data concerning mortality in geriatric patients assessed over a more prolonged period (i.e., beyond 1 y) following hospital admission. Moreover, it is unclear whether the different sarcopenia definitions affect the relation with mortality, and/or which characteristics of sarcopenia may best explain the proposed association with mortality.

Therefore the present study evaluates whether sarcopenia according to the criteria of the EWGSOP, IWGS, SIG and FNIH is associated with mortality in acutely hospitalized geriatric patients. Secondly, we determined which hallmarks of sarcopenia and/or other patient characteristics can best predict mortality in geriatric patients admitted to the acute geriatric ward.

## Methods

### Study sample

Geriatric patients admitted to the acute geriatric ward of a Dutch general hospital were asked to participate in the study. The inclusion criteria were: age above 70 y, the ability to walk prior the onset of the acute illness leading to hospital admission, being frail according to the Groningen Frailty Indicator (GFI) (17) and if there was a written informed consent obtained from the patient or proxy. Patients were excluded if they had a pacemaker or an implantable cardioverter defibrillator (ICD) because of bio impedance measurement, were not able to follow instructions because of a severe delirium or dementia, or had a terminal condition. Further details of patient selection, inand exclusion criteria and patient characteristics are described in our earlier publication (9). Measurement of gait speed, handgrip strength and body composition was done within four days after hospitalization. All patients were informed on the nature of the measurements before written informed consent was obtained from the patient or proxy. This study complied with the guidelines set out in the Declaration of Helsinki and was approved by the Ethics Committee of Sittard-Heerlen, the Netherlands (number 13-N-60). From 128 eligible hospitalized geriatric patients, 47 patients were excluded (n=38 incomplete data for sarcopenia assessment, n=9 technical problems with bio-impedance or handgrip assessment), leaving a total of 81 patients included in this study.

Relevant patient characteristics were retrieved from the medical and nursing files. These included sex, age, living situation, diagnosed medical conditions, medical history and activities of daily living prior to the acute illness that led to hospital admission. Frailty was assessed using the GFI and Fried criteria. The GFI assesses the loss of functions and resources using 15 items divided over the physical, cognitive, social, and psychological domain. A score of 1 for an item indicates a problem and a total score of 4 or higher indicates frailty (17). The Fried criteria assess physical frailty based on 5 items: unintentional weight loss, weakness, self-reported exhaustion, slow walking speed, and low physical activity; a score of 3 or higher indicates physical frailty (18). Height was estimated to the nearest cm by measuring ulna length because many patients were temporarily bedridden (19). Bodyweight was measured to the nearest 0.1 kg on a sitting weight scale (SECA, Model 959). Several standard medical questionnaires and valid scales like, cumulative illness rating scale (CIRS), Short Nutritional Assessment Questionnaire (SNAQ), Katz ADL-6 and Mini Mental State Examination (MMSE) were included because of possible associations with mortality. For the 81 patients included, GFI data were missing in 2 patients, SNAQ data were missing in 2 patients, Katz-ADL data were missing in 2 patients and MMSE data were missing in 12 patients.

### Body composition measurement

The Maltron BioScan 920-II, a multi-frequency multisegmental bio-impedance (mf-ms BIA) device, was used to measure skeletal muscle mass (SMM), fat mass (FM), body cell mass (BCM) and phase angle (PA). The Maltron Bioscan 920-II has been validated for the assessment of whole body composition and segmental lean mass in elderly people (20). Phase angle has been suggested as a variable of interest from bioelectrical impedance analysis given that this variable is independent of body height and weight. It is calculated from the directly measured resistance and reactance and is associated with membrane structure and function and it is an indicator of tissue hydration and nutritional status (21). The Maltron BioScan 920-II has an eight-point electrode system, which separately measures impedance of the patient’s trunk, arms and legs at four different frequencies (5 kHz, 50 kHz 100 Hz and 200 Hz) for each body segment. Absolute skeletal muscle mass (SMM) is calculated according to the devicespecific calculation called the Maltron calculation (22). Patients were measured early in the morning before breakfast, wearing only the pyjamas, as described in the user’s manual. Absolute skeletal muscle mass (kg) was converted to skeletal muscle index (SMI) standardizing by meters squared (kg/m^2^). Likewise, relative muscle mass (RMM, %) was calculated by dividing SMM by body weight) (1). Additionally, fat mass index (FMI: FM/height2) was calculated.

### Physical performance tests

The Jamar dynamometer (Sammons Preston, Inc., Bolingbrook, IL, USA) was introduced by Bechtol (23) and is a frequently used and validated device for assessing handgrip strength in healthy elderly people. Calibration of the Jamar dynamometer was performed before, during and after cessation of the study according to the guidelines set by the manufacturer. Handgrip strength was assessed with the second handle position of the Jamar dynamometer. We applied the Southampton protocol (24). Three grip strength measurements were performed for the dominant hand, with a rest period of 30 s between successive attempts. All measurements were performed between 11 am and 2 pm. Hand dominance was estimated with Edinburgh handedness inventory (25). Maximal handgrip strength was used as marker of muscle strength in the geriatric patients.

The Short Physical Performance Battery (SPPB) and the Hierarchical Balance and Mobility (HABAM) were used to assess mobility. The SPPB consists of 3 parts: balance, gait speed and chair stand test, each scored with a maximum of 4 points. Hence, the total range is from 0 to 12 points (26) with the highest score representing the best performance. Volpato et al. showed that the SPPB has predictive value for functional decline and mortality in hospitalized elderly (27). The HABAM was developed in the 1990’s, by MacKnight and Rockwood, and provides information about balance, transfers and mobility. The higher the score the better the mobility level, with scores ranging from 0–67 (28). Hubbard et al. showed that the HABAM provides useful information about disease progression in hospitalized elderly (29). Gait speed was assessed according the 4-meter walking test. The four-meter walking test has been validated in elderly people. The faster of two trials was used and the test was started from a standing still position. Patients were instructed to walk at an easy usual speed and were allowed to use a walking aid if necessary (9). A total of 20 patients were unable to walk at hospital admission due to the acute illness. In these patients, gait speed was assessed after 1 week. Classification for ‘sarcopenia’ vs ‘no sarcopenia’ (see below) was not affected by the initial lack of gait speed data (i.e., all 20 patients walked slower than 0.8 m/s).

### Criteria for sarcopenia

Table 1 shows the different diagnostic criteria we applied, with cut-off values for sarcopenia according to four consensus groups: EWGSOP, IWGS, SIG and FNIH. The FNIH uses appendicular skeletal muscle (aSM) mass as a criterion. However aSM mass data were only available for a limited number of geriatric patients. To our knowledge there are no specific publications concerning the validation of the use of total skeletal muscle mass to replace appendicular skeletal muscle mass. However, it is known that 73-75% of total skeletal muscle mass consist of appendicular skeletal muscle mass (30). Therefore we alternatively applied the BIA criteria for low SMI based on 2 SD below mean of young adults (1, 31), since these data were available for the majority of our population. Likewise, muscle mass criteria for IWGS were originally based on DXA criteria, but instead we applied the BIA cut off values for SMI according the NHANES III muscle mass criteria (1). All criteria used to determine whether a patient was sarcopenic or non-sarcopenic were based on measurements of muscle mass by BIA and gait speed using the 4-meter walking test. Additionally handgrip strength by Jamar dynamometer was utilized for EWGSOP and FNIH criteria.

### Patient status 2 years after hospitalization

Information on each patient’s status was retrieved from the hospital electronic medical file, primary care physician and/ or patient’s primary caregiver, to determine whether patients were still alive or were deceased at 12 and 24 months after the initial hospital admission, including the exact date of death for survival curve analyses. One researcher retrieved all the information at one time point 2 years after the inclusion of the last patient.

#### Statistics

Data were analysed using SPSS Statistics version 23 (IBM Corp., Armonk, NY, USA). Patients’ characteristics are described by mean±SD and range for continuous variables and by frequencies and percentages for the categorical variables. Kaplan Meyer curves and Cox proportional hazard ratio analysis were used to assess the proportional risk of mortality after 1- and 2-years for patients with sarcopenia compared to non sarcopenic patients based on the cut-off points of sarcopenia according the consensus criteria of the EWGSOP, IWGS, SIG and FNIH.

A 2-factor ANOVA model (gender x patient status) was used to assess gender-specific differences between 1- and 2-year survivors and non-survivors with respect to: BMI, FFM, SMI, RMM, PA, BCM, FMI, SMM/FM, HGS assessed with Jamar dynamometer, GS, SPPB, HABAM, Fried score, GFI, SNAQ score, Katz-ADL score, CIRS score, MMSE and age. Subsequently, Cox proportional hazard ratio analysis was performed to determine which of these variables could best predict 1- and 2-year mortality. Finally, Cox proportional hazard ratio analysis was performed, combining consensus criteria for sarcopenia with the separate patient characteristics to determine whether the calculated hazard ratios are affected (confounded) by different covariates. Because of the limited number of patients included and the limited number of ‘events’ (i.e., number of deaths throughout the follow up period), a maximum of 3 covariates were tested at the same time. Since no major differences were observed for all analyses in relation to 1- vs 2-year mortality, we focus on presenting 2-year mortality data, referring to supplementary tables for 1-year mortality data for completeness.

## Results

### Patient characteristics

Mean age of the 81 patients included in this study was 84±5 y and 73% (n=59) were female. Seventy eight percent of the patients lived in the community, in the surroundings of the hospital. The CIRS score was 20.0±5.5; a list of main diagnosis at hospital admission and a list of the main co-morbidities present is provided in Supplementary table 1 and 2. Thirty-nine percent of the participants were malnourished, with SNAQ scores of 3 or higher. Forty-seven percent were highly ADL dependent, with a Katz ADL-6 score of 5 or 6.

**Table 1:** Criteria for low muscle mass, handgrip strength and sarcopenia adapted from the 4 consensus groups (EWGSOP, IWGS, SIG, FNIH)

**Definition**	**Criteria**	**Men**	**Women**	**Ref**
EWGSOP	-Handgrip strength (per BMI category)	≤24: ≤29kg	≤23: ≤17kg	(1, 31)
		24.1-26: ≤30kg	23.1-26: ≤17.3kg	
		26.1-28: ≤30kg	26.1-29: ≤18kg	
		>28: ≤32kg	>29: ≤21kg	
	-Gait speed	≤0.8m/s	≤0.8m/s	
	-SMI	<8.87kg/m^2^	<6.42kg/m^2^	
IWGS	Gait speed <1m/s + low SMI	<10.76kg/m^2^	<6.76 kg/m^2^	(15)
SIG	Gait speed < 0.8 m/s + low RMM	Class 1 (<37%)	Class 1 (<28%)	(45)
		Class 2 (<31%)	Class 2 (<22%)	
FNIH	Weakness + low SMI			(6)
	- Handgrip strength	<26kg	<16kg	
	- SMI	<8.87kg/m^2^	<6.42kg/m^2^	

EWGSOP=European Working Group on Sarcopenia in Older People; IWGS= International Working Group on Sarcopenia; SIG= Special Interest Group of Sarcopenia, Cachexia and Wasting Disorders; FNIH= Foundation for the National Institutes of Health; BMI: Body Mass Index; SMI=absolute skeletal muscle mass /height2; RMM= absolute skeletal muscle mass /weight

**Table 2:** Case summary of sarcopenia according to EWGSOP, IWGS, SIG, FNIH consensus criteria in acutely hospitalized geriatric patients (n=81) and 2-year mortality

	**2-year**			
	**Non-sarcopenic**		**Sarcopenic**	
	**Alive**	**Dead**	**Alive**	**Dead**
EWGSOP	30 (75%)	10 (25%)	11 (27%)	30 (73%)
IWGS	14 (64%)	8 (36%)	27 (46%)	32 (54%)
SIG	8 (32%)	17 (68%)	33 (59%)	23 (41%)
FNIH	38 (64%)	21 (36%)	3 (14%)	19 (86%)

Data represent the absolute number (and the %) of patients who deceased and were alive after 2 years according to EWGSOP, IWGS, SIG, FNIH consensus criteria of sarcopenia. EWGSOP=European Working Group on Sarcopenia in Older People; IWGS= International Working Group on Sarcopenia; SIG= Special Interest Group of Sarcopenia, Cachexia and Wasting Disorders; FNIH= Foundation for the National Institutes of Health

### Consensus criteria and mortality

The frequency of patients with sarcopenia was different using the different consensus criteria (table 1 and 2). According to the EWGSOP, IWGS, SIG and FNIH the prevalence of sarcopenia in the acutely hospitalized geriatric patients was respectively 51, 73, 69, and 27%.

**Table 3:** Cox proportional hazard ratio of 2-y mortality for acutely hospitalized geriatric patients with sarcopenia vs. no sarcopenia according the EWGSOP, IWGS, SIG and FNIH criteria for sarcopenia (n=81)

	**no sarcopenia**	**sarcopenia**	**HR**	**CI -95%**	**P**
EWGSOP	40	41	4.310	2.092-8.850	<0.001*
IWGS	22	59	1.754	0.808-3.817	0.155
SIG	25	56	0.488	0.260-0.916	0.025*
FNIH	59	22	3.571	1.901-6.711	<0.001*

Data represent the Cox proportional hazard ratio of mortality in acutely hospitalized geriatric patients with sarcopenia compared with no sarcopenia after 2 years according to EWGSOP, IWGS, SIG, FNIH consensus criteria of sarcopenia; EWGSOP=European Working Group on Sarcopenia in Older People; IWGS= International Working Group on Sarcopenia; SIG= Special Interest Group of Sarcopenia, Cachexia and Wasting Disorders; FNIH= Foundation for the National Institutes of Health; *significantly different hazard ratio between patients with or without sarcopenia

The Kaplan Meyer survival curves showed significantly higher mortality rates for the sarcopenic compared with nonsarcopenic acutely hospitalized geriatric patients according to the consensus criteria of the EWGSOP (73% versus 25% 2-year mortality; Figure 1A) and FNIH (86% versus 36% 2-year mortality; Figure 1B), but not for the IWGS and SIG (Table 2). Remarkably, 2-year mortality was higher in the non-sarcopenic compared with the sarcopenic patients according to the SIG criteria (68% versus 41%). In agreement with the Kaplan Meyer curves, hazard ratios for mortality were significantly higher in sarcopenic patients compared to non-sarcopenic patients when using the EWGSOP (2-y HR 4.310; CI-95%: 2.092-8.850; P<l0.001; Figure 2A) and FNIH criteria (2-y HR 3.571; CI-95%: 1.901-6.711; P<0.001; Figure 2B) but not for IWGS, while the SIG criteria showed a reduced 2-y HR for sarcopenic vs nonsarcopenic patients (Table 3). Data for 1 year mortality were similar and are provided in Supplementary tables 3–4.

### Body composition parameters and 2-year mortality

To assess the association of various characteristics, both sarcopenia-related and not sarcopenia-related, with mortality, comparisons were made between patients that did and did not survive after 2 years (Table 4). The geriatric patients who deceased within 2 years after initial hospitalization were significantly (P<0.05) older, and had a lower BMI compared to the patients who were still alive. The Relative Muscle Mass (RMM) and Fat Free Mass (FFM) were significantly lower in women vs men, but there was no difference in RMM and FFM between the patients who deceased and those that were still alive. In contrast, skeletal muscle mass index (SMI) was significantly (P<0.05) lower both in women vs men, and in the geriatric patients who deceased compared to those who survived.

**Figure 1:**
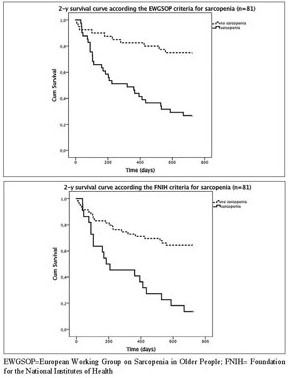
Kaplan Meier Survival curve for acutely hospitalized geriatric patients with or without sarcopenia according the EWGSOP (A; 2-year mortality, P<0.001) and FNIH (B; 2-year mortality, P<0.001)

**Figure 1:**
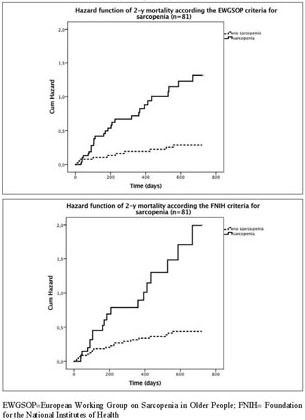
Hazard function curve of 2-y mortality for acutely hospitalized geriatric patients with or without sarcopenia according the EWGSOP (A; 2-year mortality, P<0.001) and FNIH (B; 2-year mortality, P<0.001)

Patients who deceased had a significantly lower Phase Angle (PA) compared to the patients who were alive 2 years after initial hospital admission, with no significant gender difference (2-y: PA women: 6.2±1.3 vs 7.0±1.4; PA men: 6.0±1.6 versus 7.4±1.7; all P<0.05). However the PA was higher in men who were still alive compared to women. Body Cell Mass (BCM) was significantly higher (P<0.05) in men compared to women and was significantly lower in the geriatric patients who were deceased after 2 years compared to those who survived.

In line with BMI and SMI, the fat mass index (FMI) was different between men and women, and FMI was significantly lower in the geriatric patients who had deceased after 2 years compared to the patients who were still alive (2-y: FMI women: 8.0±3.8 vs 11.2±4.3; FMI men: 5.7±2.4 vs 7.8±3.2 kg/m2; all P<0.05). Findings for fat mass percentage were in agreement with FMI. The skeletal muscle mass-fat mass ratio (SMM/FM) was significantly lower in women compared to men and was significantly higher in the patients who were deceased after 2 years compared to the patients who survived (Table 4).

**Table 4:** Body composition, muscle strength physical function, frailty, nutrition, ADL, comorbidity and age versus 2-year survival in acutely hospitalized geriatric patients (n=81)

		**Women (n=59)**				**Men (n=22)**		
	**Deceased**	**n**	**Alive**	n	**Deceased**	**n**	**Alive**	**n**
General								
Age, y	86.0±5.4	29	83.4±5.1*	30	84.8±7.4	11	82.7±6.4*	11
BMI, kg/m^2^	23.4±4.7	29	26.7±4.8*	30	23.2±4.7	11	25.9±4.0*	11
Body composition								
FFM, kg	38.1±5.9#	29	39.6±54.2#	30	48.1±7.6	11	52.4±8.4	11
SMI, kg/m^2^	6.3±0.7#	29	6.6±0.4*#	30	7.6±1.0	11	8.5±0.8*	11
RMM, %	27.6±3.8#	29	25.4±3.2#	30	34.3±3.1	11	33.3±4.0	11
Phase angle	6.2±1.3	29	7.0±1.4*	30	6.0±1.6	11	7.4±1.7*	11
BCM, kg	19.6±2.7#	29	20.5±2.0*#	30	24.6±2.8	11	27.6±3.9*	11
FMI, kg/m^2^	8.0±3.8#	29	11.2±4.3*#	30	5.7±2.4	11	7.8±3.2*	11
FM%	33.2±8.8#	29	40.6±8.1*#	30	24.3±6.0	11	30.4±7.3*	11
SMM/FM	1.0±0.4#	29	0.7±0.3*#	30	1.5±0.4	11	1.2±0.4*	11
Physical function								
HGS Jamar, kg	14.8±5.3#	29	16.4±6.0#	30	24.2±5.5	11	26.7±8.0	11
GS, m/s	0.39±0.12#	18	0.51±0.23*#	22	0.47±0.21	11	0.79±0.44*	10
SPPB	2.2±1.9#	29	3.0±2.6#	30	4.1±2.4	11	4.6±3.3	11
HABAM	35.6±11.9	29	35.4±15.5	30	39.5±10.7	11	43.9±15.0	11
Frailty, nutrition, ADL, comorbidity and cognitive function								
Fried score	4.1±0.7	29	3.8±0.6*	30	4.0±0.6	11	3.5±0.8*	11
GFI	8.2±2.8	29	7.2±2.5	28	8.2±2.6	11	6.7±2.4	11
SNAQ	2.2±1.8	29	1.5±1.5	28	1.9±1.8	11	2.1±1.3	11
Katz-ADL	3.8±2.0	29	3.4±2.2	28	3.5±2.3	11	4.0±1.5	11
CIRS	20.2±5.2	29	18.5±5.0	30	21.7±5.0	11	20.5±6.7	11
MMSE	19.1±5.6	23	20.4±5.6	24	18.1±5.2	11	21.4±5.2	11

Data are means±SD. BMI: Body Mass Index; FFM: Fat Free Mass; SMI: Skeletal Muscle Mass Index; RRM: Relative Muscle Mass; BCM: Body Cell Mass; FMI: Fat Mass Index; FM%: Fat Mass Percentage; SMM/FM: Skeletal Muscle Mass/Fat Mass; HGS Jamar: Handgrip Strength measured with Jamar dynamometer; GS: Gait Speed; SPPB: Short Physical Performance Battery; HABAM: Hierarchical Balance and Mobility; GFI: Groningen Frailty Indicator; SNAQ: Short Nutritional Assessment Questionnaire; CIRS: Cumulative Illness Rating Scale; MMSE: Minimal Mental State Examination;*significantly different from deceased (P<.05); #significantly different from men (P<.05)

### Muscle strength and physical function versus 2-year mortality

The maximal handgrip strength was significantly lower in women compared to men. Handgrip strength was not significantly different for the geriatric patients who had deceased after 2 years compared to the patients who survived (Table 4).

Gait speed (GS) was significantly lower in women compared to men. In addition, GS was significantly lower in the geriatric patients that died within 2 years compared with those patients who survived (GS women: 0.39±0.12 vs 0.51 ±0.23 m/s; GS men: 0.47±0.21 vs 0.79 ±0.44 m/s). Almost all geriatric patients (n=77) had low physical performance with a SPPB score <4. SPPB was significantly lower in women compared to men. However, there was no significant difference in physical performance according the SPPB in the patients who deceased and those who survived after 2 years. Likewise, physical performance according the HABAM was not significantly different between geriatric patients who deceased and survived within 2 years (Table 4).

### Frailty, nutrition, ADL, comorbidity and age versus 2-year mortality

The geriatric patients who deceased within 2 years were more frail according to the Fried score compared with the patients who were still alive, but this was not confirmed according to the GFI. There was no significant difference in nutritional status (SNAQ), care dependency (Katz-ADL), comorbidity (CIRS) and cognitive function (MMSE) between the geriatric patients who had deceased or were still alive after2 years (Table 4).

For all patient characteristics described above for 2 years survival, similar differences were observed between patients that had survived vs those that were deceased after 1 year (see supplementary Table 5 for details).

**Table 5:** Hazard Ratios for potential predictors for 2-y mortality in hospitalized geriatric patients (n=81) with additional analysis including gait speed (n=61)

			**2-y mortality probability**		
		**n**	**HR**	**CI-95%**	**P**
Step 1	FMI	81	0.841	0.761-0.931	0.001*
Step 2	PA		0.678	0.531-0.864	0.002*
	FMI		0.839	0.758-0928	0.001*
Step 1	FMI	61	0.826	0.729-0.936	0.003*
Step 2	PA		0.675	0.512-0.891	0.005*
	FMI		0.835	0.734-0.949	0.006*
Step 3	PA		0.712	0.532-0.954	0.023*
	FMI		0.825	0.723-0.940	0.004*
	GS		0.085	0.010-0.729	0.025*

Data represent the results of Cox proportional hazard analysis for FMI, PA and SMI for 2-year mortality in acutely hospitalized geriatric patients (n=81). Additionally Cox proportional hazard mortality analysis were performed for the patients with available gait speed at hospital admission (n=61) FMI: Fat Mass Index; PA: Phase Angle; GS: Gait Speed. *significant (P<.05)

### Cox proportional hazard ratio

Based on the ANOVA results described above, BMI, SMI, PA, BCM, FMI, SMM/FM, Fried score, and age were included as potential predictors for 2-year mortality in a Cox proportional hazard ratio model. Cox proportional hazard ratio analysis was performed on data for n=81 geriatric patients. Patients with higher PA (HR 0.678; CI-95%:0.531-0.864; P=0.002) and higher FMI (HR 0.839; CI-95%: 0.758-0.928; P=0.001) had a significantly lower mortality probability throughout the 2 year follow-up after hospital admission (Table 5).

For a subgroup of geriatric patients (n=61) gait speed was available, and was added to the Cox proportional hazard ratio analyses. Patients with higher PA (HR 0.712; CI-95%:0.532-0.954; P=0.023), higher FMI (HR 0.825; CI-95%: 0.723-0.940; P=0.004) and higher gait speed (HR 0.085; CI-95%: 0.010-0.729; P=0.025) had a significantly lower 2-y mortality probability (Table 5). Again, similar findings were observed for 1 year mortality (Supplementary Table 6).

As a final step in the analysis, we assessed whether the HRs for the presence of sarcopenia according the EWGSOP and FNIH criteria were affected by correcting for other variables. Therefore, the following patient characteristics were separately added as covariates in the Cox proportional hazard ratio analysis: age, gender, BMI, Katz-ADL, SNAQ, CIRS and MMSE. None of these variables was associated with mortality probability (HR not different from 1), and none of these variables changed the hazard ratio for the presence of sarcopenia according the EWGSOP and FNIH criteria. Only GFI was found to be independently associated with mortality probability. Therefore, in the final model, inclusion of sarcopenia criteria was combined with the inclusion of PA, FMI, and GFI, as these were all shown to be (separately) affecting the mortality hazard ratio. 2-y mortality probability was reduced in patients with higher PA (HR 0.699; CI-95%:0.546-0.895; P=0.005), and increased in patients with higher GFI (HR 1.120; CI-95%: 1.001-1.254; P=0.049), and in patients with sarcopenia according the EWGSOP criteria, with essentially unaltered HR (HR 4.040; CI-95%:1.960-8.239; P<0.001) compared to the unadjusted model (Table 3). FMI was no longer associated with 2-y mortality probability when corrected for the other variables (HR 0.924; CI-95%:0.812- 1.052; P=0.232). When the model was ran using the FNIH criteria for sarcopenia, 2-y mortality was reduced in patients with higher PA (HR 0.679; CI-95%:0.527-0.874; P=0.003), and increased in patients with sarcopenia, with essentially unaltered HR (HR 3.433; CI-95%:1.822-6.506; P<0.001) compared to the unadjusted model (Table 3).

## Discussion

In this study we demonstrate that sarcopenia was highly prevalent in older patients admitted to the acute geriatric ward, but varied widely (27-73%) when different sarcopenia criteria were used. Only sarcopenia according to the EWGSOP and the FNIH criteria was significantly associated with up to 4.3 times higher mortality probability compared to non-sarcopenic patients Additionally several hallmarks of sarcopenia and other patient characteristics, including skeletal muscle mass index, fat mass index, body cell mass, body mass index, phase angle and gait speed, were significantly lower in the geriatric patients who had deceased after 2 years compared to the patients who were still alive. Cox proportional hazard ratio analysis showed that higher gait speed, phase angle, and fat mass index are associated with reduced 2-year mortality probability in these hospitalized geriatric patients. However when correcting for various covariates, mortality probability remained strongly associated with sarcopenia according EWGSOP and FNIH criteria, with phase angle significantly adding to the model.

As expected, the prevalence of sarcopenia was high in our population of hospitalized geriatric patients. In accordance with results from the Leiden Longevity Study however, sarcopenia prevalence varied substantially when different criteria were used (32). Given the recent recognition of sarcopenia as a medical condition with its own ICD-10 CM code (M62.84), there is a clear need for well defined and generally acknowledged criteria for sarcopenia (33, 34). Indeed, to enable better comparison between studies, to specify prevalence rates, and to better target those in need of treatment, further consensus has to be reached on the exact diagnostic criteria and cut-off values for sarcopenia.

Apart from clearly establishing the diagnosis of sarcopenia, consensus criteria need to have power to predict adverse outcome like mortality. For this reason we studied the predictive value of different diagnostic criteria for sarcopenia and, subsequently, individual parameters of physical function and body composition for mortality. When applying the criteria of sarcopenia according to the different consensus groups, only the EWGSOP and FNIH criteria were significantly associated with an increased 2-year mortality in sarcopenic vs nonsarcopenic patients. Until now, mortality has only been studied up to 3–12 months after hospital admission. In accordance with our findings, Cerri and colleagues (12) previously found an increased 3-month mortality rate in hospitalized malnourished geriatric patients applying the EWGSOP algorithm. Average gait speed and SMI was higher in their study when compared to our findings. Additionally the study of Perez-Zepeda and co-workers (13) showed a comparable increased 1 year mortality in sarcopenic geriatric patients applying EWGSOP criteria. However their study population was different from our population because they excluded patients with delirium and dementia and measurement was done within 6 days after hospital admission after transfer from an acute medical unit. Besides that, cut off values for skeletal muscle mass and gait speed were different from the original EWGSOP algorithm (13). In contrast to these findings of increased mortality up to 2 years after hospitalization in sarcopenic vs non-sarcopenic geriatric patients, sarcopenic patients according to the SIG criteria had a better 2-year survival compared to the nonsarcopenic patients. One of the hallmarks in the SIG criteria is relative skeletal muscle mass (RMM), which means skeletal muscle mass divided by body mass. Low RMM can be apparent when skeletal muscle mass is normal, but body mass is (relatively) high as a consequence of increased fat mass. Likewise, ‘normal’ RMM (and thus ‘no sarcopenia’) could be associated with low skeletal muscle mass in the combination with even lower total body mass. As such, truly cachectic patients (who probably have a higher mortality) may be defined as non-sarcopenic, whereas ‘overweight’ patients with a normal muscle mass may be defined as sarcopenic when using the SIG criteria. This likely explains the contradictory relation with mortality observed in the present study. Indeed, previous work has also described a partly protective effect of minor overweight in older people (35), at least partly explaining our findings. In agreement, we show in the present study that the patients who had survived after 2 years had a higher fat mass index compared with those who had died.

Overall, the geriatric patients in our study were extremely frail, with mean handgrip strength and mean gait speed far below the cut-off values of the different consensus criteria. This homogeneity in physical performance below cut-off values likely resulted in poor discriminative potential of the sarcopenia criteria according IWGS to predict mortality within our population of frail acutely hospitalized geriatric patients.

Because of the huge differences in prevalence and difference of association of sarcopenia between the different consensus criteria and mortality, we next studied individual parameters of sarcopenia like body composition and physical function, rather than only differentiating between sarcopenic and nonsarcopenic. We show that apart from skeletal muscle mass index and gait speed (i.e., sarcopenia associated parameters), also phase angle, body cell mass, and fat mass index/percentage were significantly different between the geriatric patients who deceased and those who were alive after 2 years. Low skeletal muscle mass in combination with low handgrip strength or low gait speed has previously been associated with an increased mortality in hospitalized elderly patients (12, 36). The phase angle is a marker of overall cell and tissue vitality (37). The association between phase angle and mortality in geriatric patients is in agreement with earlier observations in cancer patients (37), as well as in a community-dwelling population of older adults (38). Also in line with our findings, Bouillanne and co-workers have shown that increased fat mass is associated with decreased adverse outcome like mortality in hospitalized elderly patients (39). In the present study, gait speed was very low and, on average, far below the cut off values of the different consensus criteria. However, when studied as an individual parameter, gait speed was still significantly lower in the geriatric patients who deceased after 2-years compared to those who survived. In acute care settings, lower gait speed (0.46 m/s) was found in patients aged 70 y and older compared with gait speed recorded in outpatient settings (0.74 m/s) (40). In agreement with earlier studies, gait speed is a strong predictor of mortality (41), however in a recent review this was only confirmed for men (42). Handgrip strength was far below the cut off point in the EWGSOP criteria but not significantly lower in the geriatric patients who had deceased after 2 years. The widely used screening tests for frailty (Fried, GFI), malnutrition (SNAQ), functional decline (Katz-ADL), comorbidity (CIRS) and cognitive function (MMSE) were not associated with mortality in this frail geriatric population. Taking these findings all together, only parameters of physical function and body composition seem to be associated with mortality in these hospitalized geriatric patients, with no major differences in their relation with 2 year mortality.

To truly determine which of the parameters that differed between survivors and non-survivors could predict mortality in these hospitalized geriatric patients we performed Cox proportional hazard ratio analysis. Based on the hazard ratio’s shown in Table 5 (and Supplementary table 6), we clearly showed that the combination of phase angle and FMI could best predict mortality risk. For example, mortality risk at any given point in time throughout the 2-yr period after hospital admission was 47.5% lower with each unit increase in phase angle, and 19.2% lower with each kg/m2 increase in fat mass. In the subgroup of patients for which gait speed data were available, mortality risk throughout the 2-yr period after hospital admission was 40.4% lower with each unit increase in phase angle, 21.2% lower with each kg/m2 increase in fat mass, and 17.6% lower for each 0.1 m/s increase in gait speed. Based on the final regression models in which we combined both sarcopenia (EWGSOP or FNIH) and the separate patient characteristics, thus correcting for several covariates, mortality probability remained strongly associated with sarcopenia, with phase angle significantly adding to the model. Though generalization of these findings should obviously be done with caution given the relatively small number of patients included in this study, our findings strongly indicate that certain physical characteristics -that are not necessarily used in the assessment of sarcopenia- are predictive for overall mortality in acutely hospitalized geriatric patients and, as such, may represent relevant diagnostic tools that may be taken into account when determining the treatment plan of these patients.

The current study was a single centre study, limited to one acute care geriatric ward of a Dutch general hospital and we only included geriatric patients who were mobile prior to hospitalization and were (cognitively) able to follow our study instructions. As such, we included a relatively small number of patients and could only adjust our analyses for a limited number of covariates. It is thus difficult to generalize our findings to the overall population of acutely hospitalized geriatric patients. Furthermore, we had missing values for 33% of the eligible patients. Although age and physically frailty in these patients was comparable to the included patients (data not shown), we cannot exclude potential confounding effects of this substantial ‘dropout’. It does however support the notion that it is extremely difficult to include these type of patients in this type of research. As a third limitation, gait speed was lacking in almost 25% (n=20) patients at hospital admission and could therefore influence sarcopenia classification. These patients were too weak to walk at hospital admission. However, we performed a 4-meter gait speed test one week later and gait speed was in all 20 patients below 0.8 m/s (data not shown). As such, risk for misclassification was minimal, as the initial lack of gait speed data did not influence classification of these patients into sarcopenic or non-sarcopenic.

As a final limitation, we only used body composition data from the BIA assessment and thus modified the original sarcopenia criteria of the IWGS and FNIH by replacing aSM by SMI. Although it is generally acknowledged that ~75% of total muscle mass consists of aSM (30, 43), and cut-offs for SMI were based on previous reports (44), the replacement of aSM with BIA-based SMI data has in itself not been validated and may have slightly impacted the sarcopenia definition. Also, bio-impedance measures such as used in our work are affected by the hydration status of patients, and changes herein (e.g. dehydration, edema) are notorious in geriatric patients. This issue is inherent to the population studied and also affects MRI or DXA based assessments. Currently, there is no valid manner to account for this potential confounding effect. In general though, prediction equations for muscle mass based on BIA have been well validated against MRI data (20, 43), supporting its use for both research and clinical practice. Moreover, in daily clinical practice of a geriatric ward, the use of BIA is much more realistic than DXA or MRI scans. Indeed, bio-impedance represents an easy accessible tool for measuring body composition with substantial predictive power for mortality, which could be of considerable value in clinical practice. This may be especially the case in targeting medical treatment in relation to geriatric patients with very limited life expectancy. Based on our findings, it could be valuable for the daily practice of a geriatrician to assess gait speed and body composition with bio impedance analysis for skeletal muscle mass, fat mass, and phase angle to identify those patients with an increased mortality risk. This may be especially relevant when a decision should be made when a medical treatment with huge impact is considered in hospitalized geriatric patients. However our study results should first be confirmed in larger clinical trials, including more centres and representing a larger spectrum of the total population of acutely hospitalized geriatric patients, also enabling the adjustment of potential relevant covariates, and including other relevant parameters such as physical functioning or readmission rates, before concrete clinical implementation is in order.

In conclusion, we show that prevalence of sarcopenia in acutely hospitalized geriatric patients is highly dependent on the criteria used. Sarcopenia according the EWGSOP and FNIH criteria is highly present and is associated with increased 2-y mortality in acutely hospitalized geriatric patients. Mortality probability is also predicted by variables like phase angle and fat mass. However when correcting for several confounders, mortality probability is best predicted by the combination of sarcopenia and phase angle. We propose that systematic bio-impedance based assessment of sarcopenia and phase angle could be of additional value in daily practice of geriatric hospital care.

*Conflicts of interest:* No conflicts of interests.

## Electronic supplementary material

Supplementary material, approximately 87.2 KB.
