# Supplementary material for: Sarcopenia is Related to Mortality in the Acutely Hospitalized Geriatric Patient
Source: J Nutr Health Aging. 2019 Jan 29;23(2):128–37. doi: 10.1007/s12603-018-1134-1 (PMC6399956; doi:10.1007/s12603-018-1134-1)
Supplement: Supplementary file 1 — Supplementary material, approximately 87.2 KB. [file mmc1.docx]

***Supplementary table 1:*** *Case summary of the 3 most frequent co-morbid diseases according CIRS category of the acutely hospitalized geriatric patients (n=81)*

| Cat. | Organ system | 1^st^ | n | 2^nd^ | n | 3^th^ | n |
| --- | --- | --- | --- | --- | --- | --- | --- |
| 1 | Cardiac | Chronic heart failure | 33 | Coronary artery disease | 13 | Atrial Fibrillation | 12 |
| 2 | Hypertension | 2 medications | 34 | 3 medications | 13 | 1 medication | 8 |
| 3 | Vascular | Anaemia | 56 | Peripheral vascular disease | 11 | DVT/PE | 8 |
| 4 | Respiratory | COPD | 34 | Pneumonia | 13 | Smoking | 11 |
| 5 | ENT | Presbyacusis | 56 | Presbyopia | 24 | Cataract | 12 |
| 6 | Upper GI | Proton Pump Inhibiter use | 25 | Reflux Esophagitis | 19 | Peptic Ulcer | 9 |
| 7 | Lower GI | Constipation | 31 | Diverticular disease | 29 | Colon cancer | 4 |
| 8 | Hepatic | Cholecystectomy | 13 | Cholecystolithiasis | 4 | Pancreatitis | 2 |
| 9 | Renal | Renal failure | 19 | Stones | 10 | Renal Cell Carcinoma | 2 |
| 10 | Other GU | Incontinence | 34 | Bladder retention | 14 | BPH/TURP | 11 |
| 11 | Musculoskeletal-Integumentary | Osteoarthritis | 36 | Fracture | 18 | Rheumatic arthritis | 11 |
| 12 | Neurological | Stroke/TIA | 34 | Parkinson(ism) | 5 | Epilepsy | 4 |
| 13 | Endocrine/ metabolic | DM | 30 | Thyroid disease | 6 | Hypercholesterolemia | 11 |
| 14 | Psychiatric/ Behavioural | Dementia | 39 | Delirium | 27 | MCI | 14 |

*CIRS: Cumulative Illness Rating Scale; ENT: Ear, Nose, Throat; GI: Gastrointestinal; COPD: Chronic Obstructive Pulmonary Disease; TIA: Transient Ischaemic Attack; DM: Diabetes Mellitus; DVT: Deep Venous Thrombosis; PE: Pulmonary Embolism; BPH: Benign Prostatic Hypertrophy; TURP: Transurethral Resection of the Prostate; MCI: Mild Cognitive Impairment*

***Supplementary table 2:*** *Case summary of main medical diagnosis at hospital admission of the acutely hospitalized geriatric patients (n=81)*

| **Main medical diagnosis** | **Number** |
| --- | --- |
| Pneumonia | 21 |
| Delirium | 15 |
| Decompensated heart failure | 7 |
| Vertebral osteoporotic fracture | 6 |
| Falls/syncope | 6 |
| Septic shock | 2 |
| Cachexia | 2 |
| Anaemia | 2 |
| Medication intoxication | 2 |
| Diverticulitis | 2 |
| Erysipelas | 2 |
| Gastric cancer | 2 |
| Other* | 12 |

** Acute cholecystitis, endocarditis, glomerulonephritis, gout arthritis, pulmonary embolism, myasthenia gravis, acute renal failure, osteomyelitis, polymyalgia rheumatic, SIADH, spondylodiscitis, subdural hematoma. All the geriatric patients had at least 5 other medical diagnosis/problems at hospital admission.*

***Supplementary table 3:*** *Case summary of sarcopenia according to EWGSOP, IWGS, SIG, FNIH consensus criteria in acutely hospitalized geriatric patients (n=81) and 1-year mortality.*

|  | **1-year** | | | |
| --- | --- | --- | --- | --- |
|  | **Non-sarcopenic** | | **Sarcopenic** | |
|  | **Alive** | **Dead** | **Alive** | **Dead** |
| **EWGSOP** | 33 (83%) | 7 (17%) | 17 (41%) | 24 (59%) |
| **IWGS** | 17 (77%) | 5 (23%) | 33 (56%) | 26 (44%) |
| **SIG** | 13 (52%) | 12 (48%) | 38 (68%) | 18 (32%) |
| **FNIH** | 42 (71%) | 17 (29%) | 8 (36%) | 14 (64%) |

*Data represent the absolute number (and the %) of patients who deceased and were alive after 1 year according to EWGSOP, IWGS, SIG, FNIH consensus criteria of sarcopenia.*

*EWGSOP=European Working Group on Sarcopenia in Older People; IWGS= International Working Group on Sarcopenia; SIG= Special Interest Group of Sarcopenia, Cachexia and Wasting Disorders; FNIH= Foundation for the National Institutes of Health*

***Supplementary table 4:*** *Cox proportional hazard ratio of 1-y mortality for acutely hospitalized geriatric patients with sarcopenia vs. no sarcopenia according the EWGSOP, IWGS, SIG and FNIH criteria for sarcopenia (n=81).*

|  | no sarcopenia | sarcopenia | HR | CI -95% | *P* |
| --- | --- | --- | --- | --- | --- |
| EWGSOP | 40 | 41 | 4.273 | 1.838-10.000 | 0.001* |
| IWGS | 22 | 59 | 2.174 | 0.835-5.681 | 0.112 |
| SIG | 25 | 56 | 0.529 | 0.259-1.082 | 0.081 |
| FNIH | 59 | 22 | 2.778 | 1.362-5.650 | 0.005* |

*Data represent the Cox proportional hazard ratio of mortality in acutely hospitalized geriatric patients with sarcopenia compared with no sarcopenia after 1 year according to EWGSOP, IWGS, SIG, FNIH consensus criteria of sarcopenia.*

*EWGSOP=European Working Group on Sarcopenia in Older People; IWGS= International Working Group on Sarcopenia; SIG= Special Interest Group of Sarcopenia, Cachexia and Wasting Disorders; FNIH= Foundation for the National Institutes of Health*

**significantly different hazard ratio between patients with or without sarcopenia*

***Supplementary Table 5:*** *Body composition, muscle strength physical function, frailty, nutrition, ADL, comorbidity and age versus 1-year survival in acutely hospitalized geriatric patients (n=81).*

|  | **Women (n=59)** | | | | **Men (n=22)** | | | |
| --- | --- | --- | --- | --- | --- | --- | --- | --- |
|  | **Deceased** | n | **Alive** | n | **Deceased** | n | **Alive** | n |
| **General** | | | | | | | | |
| **Age** | 85.2±5.1 | 22 | 84.4±5.6 | 37 | 84.4±7.4 | 9 | 81.6±6.2 | 13 |
| **BMI** | 22.9±4.9 | 22 | 26.4±4.7* | 37 | 23.2±4.2 | 9 | 25.5±4.7* | 13 |
| **Body composition** | | | | | | | | |
| **FFM, kg** | 37.6±4.5^#^ | 22 | 39.6±5.5^#^ | 37 | 48.9±6.4 | 9 | 51.2±9.3 | 13 |
| **SMI, kg/m^2^** | 6.2±0.5^#^ | 22 | 6.6±0.6*^#^ | 37 | 7.6±1.0 | 9 | 8.3±0.9* | 13 |
| **RMM, %** | 27.9±4.2^#^ | 22 | 25.6±3.1^#^ | 37 | 34.2±2.1 | 9 | 33.5±4.3 | 13 |
| **Phase angle** | 6.3±1.4 | 22 | 6.7±1.4* | 37 | 5.7±1.7 | 9 | 7.4±1.6* | 13 |
| **BCM, kg** | 19.5±2.0^#^ | 22 | 20.4±2.5*^#^ | 37 | 24.7±2.4 | 9 | 27.0±3.9* | 13 |
| **FMI, kg/m^2^** | 7.8±4.2^#^ | 22 | 10.7±4.1*^#^ | 37 | 5.3±1.7 | 9 | 7.7±3.2* | 13 |
| **FM%** | 33.0±9.3^#^ | 22 | 39.4±8.3*^#^ | 37 | 23.3±5.1 | 9 | 30.1±7.3* | 13 |
| **SMM/FM** | 1.0±0.5^#^ | 22 | 0.7±0.3*^#^ | 37 | 1.5±0.3 | 9 | 1.2±0.5* | 13 |
| **Physical function** | | | | | | | | |
| **HGS Jamar, kg** | 15.6±5.3^#^ | 22 | 15.6±5.9^#^ | 37 | 23.5±5.7 | 9 | 26.8±7.5 | 13 |
| **GS, m/s** | 0.38±0.13^#^ | 12 | 0.49±0.2*^#^ | 27 | 0.46±0.2 | 9 | 0.74±0.4* | 13 |
| **SPPB** | 2.1±2.0^#^ | 22 | 3.0±2.5^#^ | 37 | 4.2±2.4 | 9 | 4.4±3.3 | 13 |
| **HABAM** | 34.6±13.1 | 22 | 36.0±14.4 | 37 | 37.6±9.2 | 9 | 44.5±14.6 | 13 |
| **Frailty, nutrition, ADL, comorbidity and cognitive function** | | | | | | | | |
| **Fried score** | 4.1±0.7 | 22 | 3.9±0.6* | 37 | 4.1±0.6 | 9 | 3.5±0.8* | 13 |
| **GFI** | 8.4±2.5 | 22 | 7.3±2.7 | 35 | 7.7±2.5 | 9 | 7.3±2.7 | 13 |
| **SNAQ** | 2.3±1.7 | 22 | 1.6±1.6 | 35 | 1.9±1.7 | 9 | 2.1±1.4 | 13 |
| **Katz-ADL** | 3.5±2.1 | 22 | 3.6±2.2 | 35 | 3.1±2.4 | 9 | 4.1±1.5 | 13 |
| **CIRS** | 19.2±4.4 | 22 | 19.5±5.6 | 37 | 22.9±4.2 | 9 | 19.8±6.6 | 13 |
| **MMSE** | 19.4±5.4 | 18 | 19.9±5.8 | 29 | 19.2±5.0 | 9 | 20.1±5.4 | 13 |

*Data are means*±SD. *BMI: Body Mass Index; FFM: Fat Free Mass; SMI: Skeletal Muscle Mass Index; RRM: Relative Muscle Mass; BCM: Body Cell Mass; FMI: Fat Mass Index; FM%: Fat Mass Percentage; SMM/FM: Skeletal Muscle Mass/Fat Mass; HGS Jamar: Handgrip Strength measured with Jamar dynamometer; GS: Gait Speed; SPPB: Short Physical Performance Battery; HABAM:* *Hierarchical Balance and Mobility; GFI: Groningen Frailty Indicator; SNAQ: Short Nutritional Assessment Questionnaire; CIRS: Cumulative Illness Rating Scale; MMSE: Minimal Mental State Examination;*significantly different from deceased (P<.05); ^#^significantly different from men (P<.05)****.***

***Supplementary table 6****: Hazard Ratios for potential predictors for 1-y mortality in hospitalized geriatric patients (n=81) with additional analysis including gait speed (n=61)*

| **1-y mortality probability** | | | | | |
| --- | --- | --- | --- | --- | --- |
|  | n |  | HR | CI-95% | *P* |
| Step 1 | 81 | FMI | 0.835 | 0.743-0.939 | *0.003** |
| Step 2 |  | PA | 0.706 | 0.536-0.931 | *0.013** |
|  |  | FMI | 0.835 | 0.743-0.940 | *0.003** |
| Step 1 | 61 | FMI | 0.783 | 0.666-0.920 | *0.003** |
| Step 2 |  | FMI | 0.764 | 0.651-0.897 | *0.001** |
|  |  | GS | 0.030 | 0.002-0.535 | *0.017** |

*Data represent the results of Cox proportional hazard analysis for FMI, PA and SMI for 1-year mortality in acutely hospitalized geriatric patients (n=81). Additionally Cox proportional hazard mortality analysis were performed for the patients with available gait speed at hospital admission (n=61) FMI: Fat Mass Index; PA: Phase Angle; GS: Gait Speed. *significant (P<.05)*
